# Effects of Ad Libitum Feeding and Arginine Supplementation on Growth of Piglets and Reproductive Performance Parameters of Sows

**DOI:** 10.3390/ani16172687

**Published:** 2026-08-28

**Authors:** Alexander Kulüke, Cornelia Schwennen, Amr Abd El-Wahab, Christian Visscher

**Affiliations:** 1 Institute for Animal Nutrition, University of Veterinary Medicine Hannover, Foundation, Bischofsholer Damm 15, D-30173 Hannover, Germany; sekretariat-tierernaehrung@tiho-hannover.de (A.K.); amr.abd.el-wahab@tiho-hannover.de (A.A.E.-W.); christian.visscher@tiho-hannover.de (C.V.); 2 Department of Nutrition and Clinical Nutrition, Faculty of Veterinary Medicine, Mansoura University, Mansoura 35516, Egypt

**Keywords:** arginine, backfat thickness, body weight, lactating sows, reproduction parameters

## Abstract

The study evaluated the effects of ad libitum feeding with or without L-arginine (Arg) supplementation during lactation on sow performance, piglet growth, and sow backfat thickness. Seventy-one sows were assigned to three dietary treatments: restricted feeding without Arg supplementation (CON; *n* = 24), ad libitum feeding without Arg (AL; *n* = 26), and ad libitum feeding supplemented with 1% Arg (AL+Arg; *n* = 21). Dietary treatments began three days postpartum. Average daily energy intake was higher in both ad libitum groups (AL and AL+Arg) compared with the control (91.9 and 91.1, respectively vs. 63.2 MJ/sow/day). Ad libitum feeding, regardless of Arg supplementation, increased sow body weight and backfat thickness at weaning (*p* < 0.05). Piglets from both ad libitum-fed groups (AL and AL+Arg) had greater weaning weights than piglets from the control group (8.03 and 8.32, respectively vs. 7.41 kg; *p* < 0.05). Arg supplementation tended to increase total litter weaning weight compared with ad libitum feeding alone. Within the light birth weight class (≤1.00 kg), piglets from the AL+Arg group showed higher daily weight gain than those light piglets from the other treatments. In conclusion, ad libitum feeding during lactation improved sow body condition and piglet growth, while Arg supplementation may be particularly beneficial for improving growth performance of low birth weight piglets.

## Simple Summary

Sows’ reproductive performance has increased markedly within the last few years. Therefore, sows must be appropriately supplied with nutrients to enhance reproductive performance. Dietary supplementation with L-arginine (Arg) has been considered an option to improve nursing performance in reproductive sows. The results of this study showed that feeding sows ad libitum during the lactation period can decrease the loss of body weight and backfat thickness compared to restricted feeding. Feeding sows ad libitum with or without Arg did not result in statistically significant differences in reproduction performance (liveborn, stillborn, etc.) of sows. Ad libitum feeding with 1% Arg supplementation significantly improved daily weight gain among low-birth-weight piglets (≤1.00 kg), although its effect on total litter weaning weight was not statistically significant.

## 1. Introduction

Ensuring an adequate supply of energy and nutrients for modern high-performance sows remains a challenge in animal nutrition [1]. Lactation represents a catabolic state in sows due to their substantially increased energy and nutrient requirements, particularly when feed intake does not increase sufficiently to meet the rising demands for milk production [2]. An adequate supply of amino acids and energy is essential for the development of the mammary gland and the initiation of milk production [1,3,4]. Insufficient feed intake leads to the mobilization of body protein and energy reserves, negatively affecting the physical condition of sows [5,6]. Currently there are different varieties of feeding regimens used for sows during lactation, ranging from restricted feeding (a set amount of feed at set feeding times) to ad libitum (constant access to feed throughout 24 h). Previous studies have shown that ad libitum feeding attenuates the loss of body reserves during lactation [7,8], increases time standing and feeding [9], and increases feed intake [8,10,11]. Mechanical sow-controlled ad libitum feeding systems were initially designed to boost feed intake during lactation by requiring sows to dispense own feed manually. However, these systems cannot track the quantity of feed consumed or the timing of feed requests. In contrast, modern computer-monitored ad libitum feeding systems use electronic sensors triggered by the sows to access feed. These advanced systems allow sows to eat whenever they choose while accurately recording feed quantities and timings. These data offer farmers exceptional management tools to monitor and adjust the sow’s feed ration throughout lactation [12].

The birth weight and uniformity of litter size at birth are crucial production variables in commercial swine production [13], as they strongly correlate with postnatal growth rate, piglet survival during lactation, and muscle development at the time of slaughtering [14]. In recent decades, various nutritional strategies have been explored to improve piglet birth weight and optimize reproductive performance in sows [15]. These strategies include the manipulation of maternal energy and nutrient supply during late gestation, as well as targeted modification of dietary protein composition through supplementation with functional amino acids such as arginine (Arg). Te Pas et al. [16] reported that gestational feeding strategies affected sow performance, behavior, and piglet outcomes. In their study, sows offered ad libitum access to a low-energy diet showed greater feed intake, body weight (BW) gain, and backfat gain than restricted-fed sows. In addition, piglets born to ad libitum-fed sows showed improved overall performance.

Arginine is an essential and a functional amino acid with multiple metabolic functions that regulate metabolic pathways via cell signaling, including phosphorylation of master regulator of intracellular protein synthesis [17,18]. Arginine is also a precursor of molecules that improve nutrient and oxygen flow from the sow to the fetus, thus contributing to fetal development and growth [19,20]. Moreover, Arg is a precursor of biologically active molecules, such as nitric oxide and polyamines, key factors in angiogenesis and placental growth [21]. Therefore, dietary supplementation with Arg has been evaluated as a potential alternative to improve fetal muscle development in pigs [22,23]. Previous studies have demonstrated that Arg supplementation alters the expression of myogenic regulatory factors and insulin-like growth factors in skeletal muscle by changing protein synthesis, cell proliferation, differentiation, and growth [24,25,26]. Arginine contributes to blood vessel formation through the production of nitric oxide, which can affect the production capacity and milk composition in lactating sows [27]. This effect arises from enhanced blood flow in the mammary gland, potentially increasing the availability of nutrients necessary for milk synthesis and its components, as well as for protein synthesis within mammary epithelial cells [28]. Furthermore, Arg may increase circulating levels of prolactin, insulin-like growth factors, and insulin in sows [29]. These are important hormones in mammary gland tissue proliferation, differentiation, and nutrient uptake.

Although Arg is a nutritionally non-essential amino acid in adult pigs, it becomes conditionally essential during periods of rapid growth and high physiological demand, such as in piglets and lactating sows [30]. Arg deficiency—an insufficient supply relative to metabolic demand—can arise from low dietary Arg, reduced intestinal citrulline synthesis (a key Arg precursor and major growth-limiting factor in young pigs) [17], inherited deficiencies of Arg-synthetic enzymes, impaired intestinal transport of Arg, and/or impaired conversion of citrulline into Arg in the kidneys [31,32]. In piglets, the condition is worsened by the low Arg content in sow’s milk, high growth requirements, and limited endogenous Arg synthesis during weaning [30,33]. Consequences of Arg deficiency in swine include growth retardation, impaired gut development and intestinal dysfunction, immune and neurological dysfunction, reproductive dysfunction, cardiovascular and pulmonary abnormalities, impaired wound healing, and hyperammonemia, with severe cases leading to death [30,34]. In sows specifically, inadequate amino acid intake during late gestation and lactation reduces productivity and longevity [35,36].

The relative contributions of sow’s milk vs. endogenous synthesis to meeting the Arg requirements of suckling piglets can be estimated on the basis of Arg intake and Arg accretion plus catabolism in the body [37]. An Arg deficiency has been identified as a major factor limiting weight gain in milk-fed piglets, as sow’s milk provides at most only 40% of the daily Arg requirement in seven-day-old suckling pigs [31]. Thus, the effects of Arg supplementation in the lactating sow diet on piglet BW and homogeneity at weaning that are still evident two weeks after weaning are the most promising for the development of new feeding strategies [38].

This study’s hypothesis was that, as opposed to a manually controlled feeding regimen, offering sows’ ad libitum access or a combination of ad libitum offer and higher dietary Arg levels during lactation might promote piglets’ development and prevent sows’ excessive body substance losses during lactation. The a priori primary endpoints were sow body weight and backfat thickness loss during lactation, and piglet body weight at weaning. Secondary endpoints included sow feed and nutrient intake, reproductive performance parameters (total born, liveborn, and stillborn piglets, in both the current and subsequent litter), and piglet daily weight gain stratified by birth weight class.

## 2. Materials and Methods

This study protocol was reviewed and approved by the Animal Welfare Officer of the University of Veterinary Medicine Hannover, Foundation, Hannover, Germany in accordance with the German protocol § 7 of the Animal Protection Law prior to conducting this study in 2016–2017 (approval number TVO-2015-V-34).

### 2.1. Farms and Animals

The farm housed 120 sows and operated with a 2.5-week farrowing rhythm and a four-week suckling period, resulting in two parallel production lines with staggered batch groups. One week prior to the expected farrowing date, sows were moved into the farrowing unit. The herd included sows of varying parity and age. The farrowing crates were equipped with piglet protection rails to reduce the risk of piglet crushing during the farrowing and early lactation period. Starting on the third day postpartum, sows were systematically assigned to one of three experimental treatments. A total of 71 sows were included in the study and assigned to the three dietary treatments: restricted feeding without Arg supplementation (CON; *n* = 24), ad libitum feeding without Arg (AL; *n* = 26), and ad libitum feeding supplemented with 1% Arg (AL+Arg; *n* = 21). Because treatments were implemented sequentially across farrowing batches, allocation was not randomized at the individual-sow level; sows were included consecutively within treatment batches, with all sows in a given batch receiving the same treatment. As a result, treatment effects cannot be fully separated from potential batch-level effects (e.g., season, staff, or environmental conditions), representing a structural limitation of the study design that should be considered when interpreting the treatment differences reported below.

### 2.2. Experimental Diet

When the sows were placed in the farrowing pen, they continued to receive the gestation diet. Starting on the third day postpartum, the sows received the lactation diet corresponding to their assigned treatment group. The experimental groups were successively moved into the farrowing pen, with an interval of approximately 2.5 weeks between groups, reflecting the two-batch sow management system of the farm. The dietary treatments (barley-soybean meal-based diet) had different feeding systems as well as different levels of Arg content. For the sows in the first group (CON), a manually controlled, established restricted feeding scheme was followed (once a day in the morning), without any Arg supplementation (AL). Feed allowance was gradually increased from day 2 to day 12 postpartum, reaching a maximum of 6 kg of lactation feed per day, without any Arg supplementation (AL). In the second group, the sows were fed ad libitum by using a special feeding dispenser (Model Sow Max^®^, Hog Slat, Inc., Newton Grove, NC, USA), whereby 2 kg feed was provided manually in the morning and the remainder dispensed automatically, without any Arg supplementation (AL). Sows in the third group were fed ad libitum using the same feeding technique as in group 2, but received a diet supplemented with 1% Arg-HCl (AL+Arg). The composition of the experimental lactation diets is shown in Table 1. Detailed vitamin and mineral composition of the premixes included in the lactating and pregnant diets is presented in Supplementary Table S1.

The chemical composition of the experimental diets in CON, AL, and AL+Arg is presented in Table 2. The metabolizable energy (per kg diet as fed) of diets CON, AL, and AL+Arg were 12.9 MJ, 12.9 MJ, and 13.2 MJ, respectively. The sows in all groups received the lactation-diet from 3 days postpartum until weaning.

The Arg (Daesang CO., Seoul, Republic of Korea) was supplied as a powder and mixed into the supplementary feed. The nutrients in the experimental diets met the nutrient requirements of the German Society for Nutrition Physiology [39]. During the study, the control and experimental feeds were analyzed regularly. Feed samples from the control and experimental diets were collected regularly and retained for later analysis. Prior to chemical analysis, individual samples were pooled to obtain representative composite samples of each diet. From each composite sample, a 100 g subsample was prepared using a sample divider for laboratory analysis. Furthermore, the diets were analyzed for crude nutrients using Weender analysis in accordance with the official methods of the Association of Agricultural Research and Testing Institutes (Verband Landwirtschaftlicher Untersuchungs- und Forschungsanstalten (VDLUFA)) [40]. In addition, the starch and mineral contents in the diets were determined. The calcium content was determined using atomic absorption spectrometry (Unicam SOLAAR M Series Flame and Furnace Atomic Absorption Spectrometer Systems, Thermo Elemental/Thermo Fisher Scientific Ltd., Cambridge, UK), in accordance with Slavin et al. [41]. A photometric characterization (UV—Visible Recording Spectrophotometer UV 162, Shimadzu Corporation, Kyoto, Japan; Wavelength 356 nm) of the phosphorus content was based on the vanadate-molybdate method, in accordance with Gericke and Kurmies [42]. Finally, the levels of amino acids were determined using LC 3000 Amino Acid Analyzer, formerly Biotronik GmbH, Maintal, Germany.

To calculate the energy content (metabolizable energy) of the feed, the results of the Weender analysis (protein, fat, fiber, ash) in addition to starch were used. The analyzed and calculated chemical composition of the experimental diets is presented in Table 2 and Table 3.

Although the CON and AL diets shared the same nominal formulation (without Arg supplementation), they were produced using on-farm mixing equipment; variation in the mixing process, feed sampling, and laboratory analysis may therefore account for minor differences in analyzed nutrient and amino acid content between these two diets (Table 3).

### 2.3. Body Weight, Backfat Thickness, Lactation Feed Intake and Calculation of Milk Production

**Figure 1:**
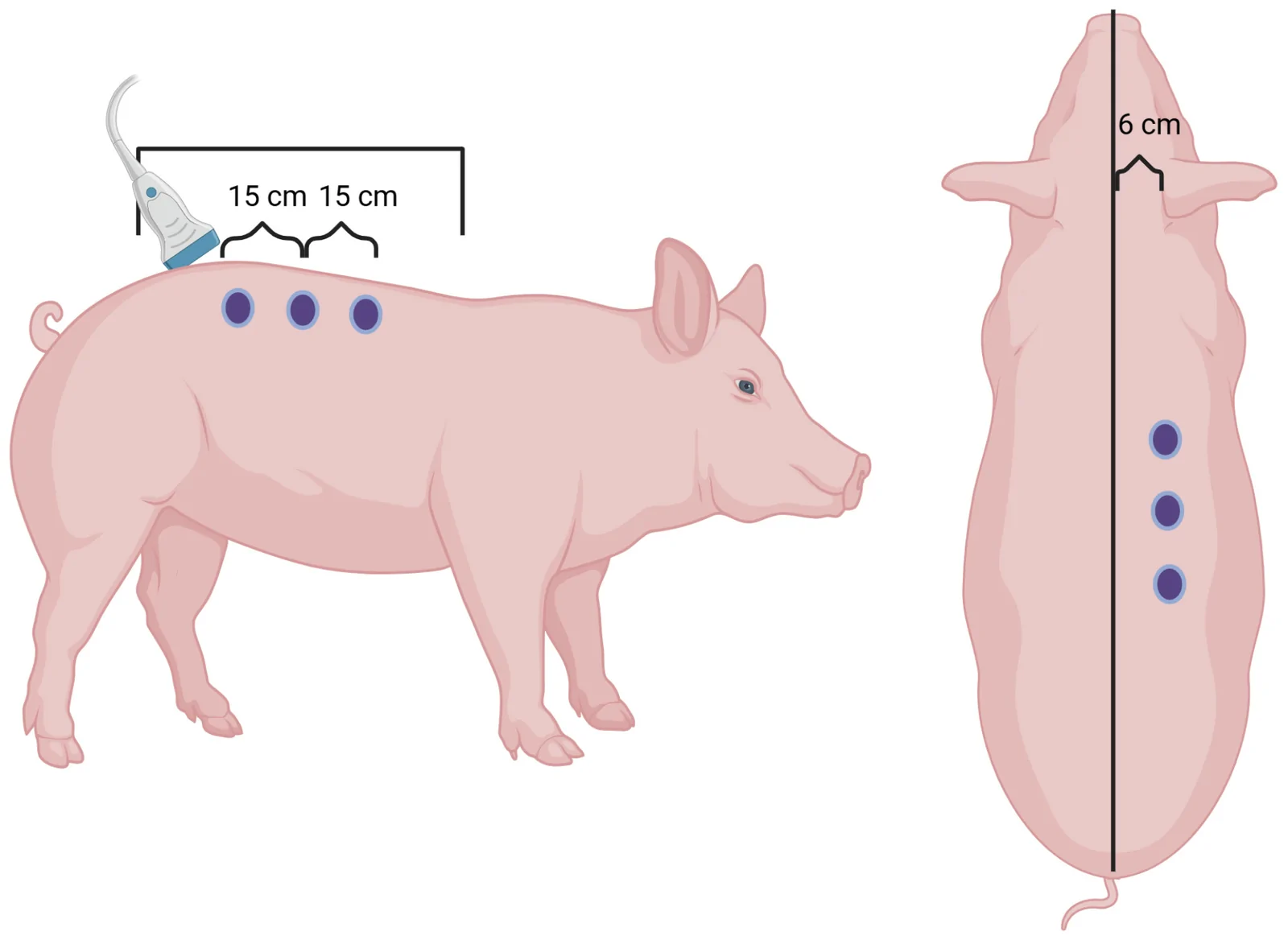
Measuring backfat thickness using Lean-Meater^®^. Created in BioRender. Visscher, C. (2026) https://BioRender.com/v86o075.

The BW and backfat thickness of each sow were measured at day −7 and 3 days before weaning. Piglets’ BW was recorded on day 1 (within 24 h after birth) and on day 26 (one day before weaning). Sow BW was measured using electric scales (Model Dr. Tenhündfeld; T.E.L.L. Steuerungssysteme GmbH & Co. KG, Vreden, Germany) for sows, and backfat thickness was measured using an ultrasound device (Lean Meter^®^, Renco Corp., Golden Valley, MN, USA) by means of a three-point measurement method according to Müller and Polten [43] (Figure 1). Sows were weighed seven days prior to farrowing and three days before weaning. As sows could not be weighed immediately after farrowing, lactational BW loss was estimated using a corrected approach. The BW measured seven days before farrowing was adjusted for the estimated weight of the litter and the associated fetal annexes. For this correction, the total birth weight of liveborn and stillborn piglets was considered, and the weight of the fetal annexes was estimated based on published data, assuming approximately 200 g of placental material per kg of newborn piglet body weight [44]. As the placental weight coefficient (200 g/kg) was derived from published estimates rather than direct measurement, corrected BW loss values should be interpreted as approximations. Variation in placental weight across sows is a source of potential error in these estimates. The corrected lactational BW loss was then calculated as the difference between the estimated postpartum BW and the BW recorded three days before weaning. The daily feed wastage was recorded during lactation, and the lactation feed intake was measured. The control group was fed according to a feeding regimen established on the farm for lactating sows and routinely applied under practical conditions. Feed was offered once daily following a predetermined feeding curve.

Sow milk production during lactation was calculated indirectly from litter weight gain. According to the German Society for Nutrition Physiology [39], 4.1 kg of sow’s milk is required per kg of litter weight gain, largely independent of litter size. As piglets in the present study also received supplemental prestarter feed, its contribution to litter weight gain was accounted for in the calculation. Using a partial efficiency of utilization of k = 0.7 for the prestarter feed, the energy intake from prestarter consumption was calculated and subtracted from the energy required for litter growth via milk. Based on a metabolizability of sow’s milk of 0.9 and a partial efficiency of utilization of 0.7, 18 MJ of sow’s milk was required to provide the energy equivalent of 1 kg of prestarter feed; given a mean energy content of 5.0 MJ/kg prestarter, 1 kg of prestarter feed was considered to replace 3.6 kg of milk. Prestarter intake was accounted for in the milk production calculation according to German Society for Nutrition Physiology [39].

### 2.4. Statistical Analysis

Statistical analysis was performed in collaboration with the Institute of Biometry, Epidemiology, and Information Processing, University of Veterinary Medicine Hannover. Data analyses were performed using SAS Enterprise Guide (version 7.1; SAS Institute Inc., Cary, NC, USA). The individual sow was the unit of measurement for feed intake and body condition parameters. However, as treatments were applied at the batch level rather than randomized within batch, sow-level measurements are not fully statistically independent of batch; this is addressed as a study limitation in Section 2.1. Normality of residuals was assessed using the Kolmogorov–Smirnov test. For normally distributed data, a one-way analysis of variance (ANOVA) followed by multiple pairwise comparisons using the Ryan–Einot–Gabriel–Welsch multiple range test was applied. For non-normally distributed data, pairwise comparisons were performed using the Wilcoxon rank sum test. Exact *p*-values were not retained; significance is therefore reported using the threshold of *p* < 0.05 as originally applied. A significance level of *p* < 0.05 was considered statistically significant. In tables, different superscript letters indicate significant differences: lowercase letters (a, b, c) denote differences within a row, whereas uppercase letters (A, B, C) denote differences within a column.

## 3. Results

The data collection took place over a period of nine months (September through May). Data were collected from a total of 71 sows, with 24 animals assigned to the control group, 26 to the AL group, and 21 to the AL+Arg group.

Providing lactating sows with ad libitum access to feed (AL and AL+Arg) resulted in significantly higher feed intake compared with the CON group during the first week of lactation (*p* < 0.05). From the second week until weaning, feed intake did not differ significantly between the ad libitum-fed groups (AL vs. AL+Arg), although both remained significantly higher than in the CON group (Table 4).

The energy and nutrient intake of sows during lactation are shown in Table 5. Sows in the CON group had the lowest energy, crude protein (CP), and crude fat intake among the experimental groups (*p* < 0.05). Significant differences in energy, crude protein, and crude fat intake were also observed between the AL and AL+Arg groups.

At weaning, sows in the CON group had the lowest BW among the experimental groups (*p* < 0.05). Corrected BW loss during lactation, calculated as described under 2.3, averaged 26.3 ± 15.1 kg in the CON group. Sows in the AL group lost 11.8 ± 16.9 kg, while sows in the AL+Arg group lost 7.0 ± 16.3 kg. Since BW was recorded before farrowing and again before weaning, parturition-associated losses such as piglets, placenta, and fetal fluids are inherently accounted for in the measurement interval, and the calculated difference provides an estimate of maternal BW change during lactation, although it remains subject to error because postpartum weight and fetal annex weight were not measured directly.

The backfat thickness of sows at entry to the farrowing compartment did not differ among the treatment groups (Table 6). However, at weaning, sows in the CON group had the lowest backfat thickness among the experimental groups (*p* < 0.05). Sows within each group had lower (*p* < 0.05) backfat thickness at weaning in comparison to that at entry to the farrowing compartment.

The backfat thickness loss (either absolute or relative) of sows in the CON group was greater (*p* < 0.05) than sows in other treatments (AL or AL+Arg), while no significant differences were seen in the backfat thickness loss (either absolute or relative) of sows between the AL and AL+Arg groups. Calculated sow milk production during lactation did not differ significantly among groups (CON: 260 ± 43.0 kg; AL: 287 ± 57.8 kg; AL+Arg: 288 ± 73.5 kg; Table 6).

At the beginning of the trial, no statistically significant differences were detected in sow reproductive performance parameters (total born, liveborn, stillborn piglets; Table 7) among groups. Likewise, no differences were observed in the reproductive performance of the follow-up litter. The total number of piglets born was slightly lower in the AL+Arg group (13.5) compared with the control and AL groups (15.7 and 15.9, respectively). Although the difference was not statistically significant, it should be noted as a potential limitation when interpreting the effects of Arg supplementation.

The number of suckling days or of weaned piglets per sow did not differ among the treatments (Table 8). Piglets from CON sows had a significantly higher BW at birth than piglets from sows in the AL and AL+Arg groups (*p* < 0.05). However, despite their higher birth weight, piglets from CON sows had the lowest BW at weaning among the experimental groups (*p* < 0.05). In contrast, piglets from sows fed the Arg-supplemented diet ad libitum (AL+Arg) had the highest BW at weaning (*p* < 0.05), followed by piglets from sows in the AL group. However, sows fed ad libitum (AL) or with Arg supplementation (AL+Arg) diets did not affect the litter growth or the amount of prestarter consumed per litter. Pre-weaning mortality, defined as the difference between the number of piglets present in the litter after standardization and the number of piglets from that litter surviving to weaning, was calculated for each sow individually; piglets not present at the final pre-weaning weighing were confirmed deceased. Mortality did not differ significantly among groups (CON: 2.5%; AL: 7.4%; AL+Arg: 3.9%; Table 8). As litter standardization is intended to allocate piglets to sows based on factors favoring survival (e.g., piglet vitality and udder access), this management practice likely contributed to the comparatively low mortality observed across all groups.

Piglet performance analyzed by birth weight class is presented in Table 9. Within the light birth weight class (≤1 kg), piglets from the AL+Arg group exhibited higher values than those from the CON or AL groups. Within each birth weight class (>1.25–1.50 kg onward), piglets from the AL and AL+Arg groups exhibited higher values than those from the CON group. These results indicate that ad libitum feeding supplemented with 1% Arg improved piglet growth performance for light piglets (≤1.00 kg).

Feed intake also varied with backfat thickness at entry to the farrowing compartment. In the AL group, sows with backfat thickness <19 mm and 19–23 mm consumed 6.5 and 6.4 kg/day, respectively, significantly more than sows with >23 mm backfat thickness (5.3 kg/day; *p* < 0.05). Similarly, in the AL+Arg group, sows with <19 mm and 19–23 mm backfat thickness consumed 6.2 and 6.5 kg/day, respectively, compared with 5.1 kg/day for sows with >23 mm backfat thickness (*p* < 0.05; Table 10).

## 4. Discussion

Supplying large litters with adequate amounts of milk is a major challenge in modern pig production, as litter sizes continue to increase. High feed intake by lactating sows is crucial both for supporting milk production [1] and for minimizing body condition losses [45] during lactation. The present study investigated the effect of sow-controlled feed intake during lactation on the performance of sows and their piglets, evaluating whether allowing sows to regulate their own feed intake could improve body condition and piglet growth. Excessive mobilization of body reserves during lactation can negatively affect the performance of both sow and piglets [46,47,48,49].

Sows receiving ad libitum access exhibited markedly higher feed intake than restrictively fed sows, consistent with the higher energy and nutrient intake reported in Table 5. The dry matter (DM) intakes observed in the ad libitum-fed groups (6.14–6.27 kg/day) were similar to those reported by other authors (5.90 kg DM/day [50]; 6.10 kg DM/day [1]) and to the ad libitum group of Schwennen et al. [8]. Briefly, Schwennen et al. [8] observed that the mean DM intake of the ad libitum group was significantly higher in the first week of lactation and tended to be higher in the following weeks, except week 2, than in the control group. With the significantly higher feed intake after birth (week 1) and the tendency of the ad libitum-fed group to have higher feed intake during each week of lactation, a higher mean DM intake of 7.02 ± 0.81 kg DM/day tended to be achieved by these animals throughout lactation than by the restrictively fed group (6.75 ± 0.44 kg DM/day). Feeding many small meals throughout the day, as enabled by the ad libitum feeders used here, appears to stimulate sows to maximize feed intake, consistent with Wehebrink et al. [51], who reported that sows fed twice a day consumed an average of 115 kg as fed over 21 days, while sows fed four times a day consumed 120 kg and 122 kg, respectively, over the same period.

The condition of sows at the end of gestation is considered to influence feed intake during lactation. Feed intake was inversely related to backfat thickness at entry to the farrowing compartment in both the AL and AL+Arg groups (Table 10), consistent with the physiological response of lactating sows to their body condition [52]. Sows with lower backfat reserves typically increase voluntary feed intake to meet the high energy demands of lactation and minimize excessive BW loss, whereas sows with greater backfat thickness may rely more on mobilizing body reserves to support milk production. Previous studies have similarly reported lower daily feed intake during lactation in sows with greater backfat thickness at day 109 of gestation, indicating an association between body condition and voluntary feed intake [53].

Dietary amino acid supply was assessed on the basis of crude protein (CP) rather than precaecally digestible CP, as recommended by the German Society for Nutrition Physiology [39]. Although the experimental diets were formulated to provide equal proportions of digestible amino acids using standard ingredient digestibility coefficients, the CP and amino acid values reported here are as-analyzed values, which were not corrected for digestibility; such correction was not possible retrospectively without a dedicated digestibility trial. Furthermore, because the high nitrogen content of L-Arg—which contains four nitrogen atoms per molecule, compared with one or two in most other amino acids—disproportionately inflates calculated CP (via Kjeldahl N × 6.25) in the AL+Arg group (Table 3), and because analyzed nutrient values can also deviate from nominal formulation due to variation in on-farm mixing, feed sampling, and laboratory analysis, the absolute CP intake values reported here should not be interpreted as indicators of biological protein adequacy or compared quantitatively against digestible-CP-based recommendations. Individual essential amino acid concentrations (Table 3) provide a more reliable basis for evaluating nutrient adequacy in this study.

Reproductive performance in the subsequent litter did not differ significantly among treatments, although sows in the AL+Arg group numerically had fewer total born and more stillborn piglets. Various factors are known to influence stillbirth rates in sows, including larger litter size and higher parity [54]. Since dietary treatments began only after farrowing, no effect on the concurrent litter was expected. As AL+Arg sows lost the least body mass during lactation, the numerically smaller subsequent litter size is unlikely to be explained by body condition loss [55]. These findings are consistent with previous studies reporting no differences in litter performance between restrictively and ad libitum-fed sows [56,57,58,59], although some studies have reported divergent results [11]. The lack of effect of Arg supplementation on litter size may reflect the timing of supplementation (beginning only after farrowing) or that the basal diet already met sows’ Arg requirements [60].

Because sows were not weighed immediately after farrowing, the uncorrected BW change over the measurement interval included not only maternal tissue mobilization but also parturition-associated losses (piglets, placenta, fetal fluids). Corrected lactational BW loss was therefore calculated based on recorded piglet birth weights and an estimated fetal annex weight of 200 g per kg newborn piglet BW [44]. Based on this correction, lactational BW losses in the CON group averaged 26.3 kg, exceeding the 20 kg loss proposed as typical by the German Society for Nutrition Physiology [39], while AL and AL+Arg sows lost considerably less (11.8 and 7.0 kg, respectively), consistent with previous findings [8,61,62,63] and suggesting a potential sparing effect of ad libitum feeding and Arg supplementation on maternal body reserves. Arginine’s roles in nitric oxide synthesis, protein metabolism, and energy utilization may contribute to improved nutrient partitioning toward milk production while limiting excessive mobilization of adipose and lean tissue. Further studies including direct postpartum weight measurements and metabolic indicators are warranted to clarify the underlying mechanisms.

The physiological response to ad libitum vs. restricted feeding likely differs, potentially reflecting differences in feeding frequency and meal size, which are known to influence satiety regulation in pigs [64]. This, together with feeding frequency and management practices, may partly explain the greater BW and backfat losses observed under restricted feeding [45,65,66]. Backfat thickness provides a more robust indicator of body condition change than BW, which is influenced by variable components such as piglets and fluids [67], and is a well-established indicator of sow body condition [68]. Insufficient energy intake during lactation is known to induce a catabolic state, leading to increased mobilization of body reserves and impaired milk production and piglet growth [8,69,70]; ad libitum feeding in the present study more closely met the metabolic demands of lactating sows, resulting in significantly lower condition losses.

Based on the present data, keeping backfat thickness below 23 mm is recommended to maximize feed intake, consistent with Vignola [71] and with general recommendations for moderate body condition (15–26 mm) [72]. Sows in the AL+Arg group showed the lowest BW loss and highest backfat thickness at weaning; while this may protect reproductive potential, excessive backfat retention could also increase the risk of metabolic or farrowing complications in the subsequent lactation. Notably, some sows in each group showed no measurable backfat loss without an apparent effect on reproductive performance, consistent with Tummaruk [73], though the association between stable backfat and later reproductive performance remains unclear. Backfat values at weaning remained relatively high across all groups (15.2–17.9 mm), indicating that even the restrictive CON regimen did not compromise body condition to a degree raising animal welfare concerns.

A high weaning weight is important both for piglet long-term health and farm profitability, influenced by factors such as sow age, body condition, management, and feeding [74]. Dietary Arg supplementation during lactation can improve sow and piglet performance [29,75], consistent with the present findings, although other studies have failed to demonstrate such effects [76,77], possibly reflecting differences in environmental conditions, management, or parity distribution. It should also be noted that the AL+Arg diet differed from the AL diet not only in Arg content but also in crude protein and several other amino acids (Table 3); therefore, effects observed in the AL+Arg group cannot be attributed to Arg supplementation alone.

Sow milk production, calculated indirectly from litter weight gain, did not differ significantly among groups. However, milk composition and colostrum quality were not assessed in the present study, despite their mechanistic relevance to Arg’s effects on mammary function. Future studies should directly assess these parameters, alongside independently validated milk yield measurements, to clarify the physiological pathways underlying the piglet growth responses to Arg supplementation observed here, particularly in low-birth-weight piglets.

## 5. Conclusions

This study indicates that ad libitum feeding of sows during lactation may reduce body weight and backfat losses compared with restricted feeding, without affecting sow reproductive performance. Ad libitum feeding, particularly when combined with 1% Arg supplementation, significantly improved daily weight gain in low-birth-weight piglets (≤1.00 kg), while its effect on overall litter weaning body weight was not statistically significant. This targeted benefit for lighter piglets could nonetheless contribute to improved litter uniformity. These findings provide practical information to support the optimization of lactation feeding strategies in commercial sow management. Future studies should evaluate different Arg supplementation levels under practical farm conditions, and should directly assess milk yield, milk composition, and pre-weaning piglet mortality, to determine cost-effective and welfare-relevant feeding strategies for lactating sows.

## Figures and Tables

**Table 1:** Ingredients (% as fed basis) in the diets for lactating sows.

Ingredient	Pregnant Diet	Lactating Diet
Barley	70	54
Triticale	10	-
Wheat	-	14
Soybean meal	9	20
Supplementary	10 ^1^	10 ^2^
Soybean oil	1	2

^1^ Composition of the pregnant diet supplement is provided in Supplementary Table S1. The pregnant diet was fed until shortly after farrowing. ^2^ Composition of the lactating diet supplement is provided in Supplementary Table S1. Depending on group assignment, sows received either a restricted lactating diet, an ad libitum lactating diet, or an ad libitum lactating diet with additional arginine supplementation via the supplement.

**Table 2:** Chemical composition of diet for pregnant sows (g/kg as dry matter basis).

Item	Pregnant Diet
Dry matter (g/kg fresh)	879
Crude ash	59.8
Crude protein	156
Crude fat	41.2
Crude fiber	67.0
Starch	440
Calcium	10.6
Phosphorus	6.56
Metabolizable energy (MJ/kg)	13.9

**Table 3:** Chemical composition of experimental diets for lactation sows (g/kg as dry matter basis).

Item	CON ^1,4^	AL ^1,4^	AL+Arg ^1,4,5^
Dry matter (g/kg fresh)	886	883	888
Crude ash	70.2	61.1	63.5
Crude protein ^2^	190	177	209
Crude fat	57.3	58.3	51.7
Crude fiber	59.2	61.2	55.1
Starch	399	408	396
Calcium	11.5	10.2	10.9
Phosphorus	6.18	5.98	5.38
Metabolizable energy (MJ/kg)	14.5	14.5	14.8
Essential amino acids			
Glycine	8.11	8.03	8.68
Histidine	4.70	4.76	4.81
Isoleucine	7.47	7.15	7.79
Leucine	13.0	13.0	14.7
Lysine	10.2	10.6	11.6
Methionine	3.12	4.04	3.13
Phenylalanine	9.36	8.88	9.57
Threonine	6.38	9.01	8.07
Valine	9.24	8.91	9.29
Semi-essential amino acid			
Arginine ^3^	11.7	12.5	20.0
Non-essential amino acids			
Alanine	8.55	7.99	8.54
Cysteine	3.23	3.18	3.24
Aspargine	16.5	17.2	19.6
Serine	9.43	9.01	10.8
Glutamine	39.4	39.6	42.5
Tyrosine	6.20	6.25	6.88

^1^ CON: Control diet; AL: ad libitum feeding without L-arginine; AL+Arg: ad libitum feeding supplemented with 1% L-arginine. ^2^ Crude protein (CP) was calculated as N × 6.25 and the high N content of L-arginine led to an overestimation of CP in AL+Arg group. ^3^ L-arginine is recognized as a conditionally essential amino acid during lactation in sows [37]. ^4^ Although the CON and AL diets were formulated to the same nominal recipe (identical ingredient composition, without Arg supplementation), they were produced using on-farm mixing equipment. Variation in the mixing process, feed sampling, and laboratory analysis may account for the observed differences in analyzed crude protein and amino acid content (e.g., crude protein, threonine, methionine) between CON and AL, rather than an intentional formulation difference. ^5^ The AL+Arg diet also differed from the AL diet in crude protein and several amino acids beyond Arg. Because arginine contains four nitrogen atoms per molecule, more than most other amino acids, its supplementation disproportionately increases nitrogen-based crude protein calculations, independent of any change in overall protein quality. As a result, effects attributed to Arg supplementation cannot be fully isolated from these broader compositional differences between the two ad libitum diets.

**Table 4:** Feed intake of lactating sows between day of birth and day of weaning (kg dry matter/head/day).

Item	CON ^1^ (*n* = 24)	AL ^1^ (*n* = 26)	AL+Arg ^1^ (*n* = 21)
d 1–7	2.20 ^c^ ± 0.28	4.30 ^a^ ± 0.90	3.80 ^b^ ± 0.76
d 8–14	4.90 ^b^ ± 0.38	6.80 ^a^ ± 0.99	6.60 ^a^ ± 1.44
d 15–21	5.30 ^b^ ± 0.11	7.20 ^a^ ± 0.95	7.50 ^a^ ± 0.90
d 21–weaning	4.80 ^b^ ± 0.43	6.80 ^a^ ± 0.88	6.70 ^a^ ± 0.95
Total feed intake	118 ^b^ ± 15.3	171 ^a^ ± 19.4	167 ^a^ ± 30.9

^a,b,c^ Means in a row with different superscripts differ significantly (*p* < 0.05). ^1^ CON: Control diet; AL: ad libitum feeding without L-arginine; AL+Arg: ad libitum feeding supplemented with 1% L-arginine.

**Table 5:** Energy and nutrient intake of sows during lactation.

Item	CON ^1^	AL ^1^	AL+Arg ^1^
Metabolizable energy (MJ/head/day)	63.2 ^b^ ± 4.42	91.9 ^a^ ± 9.62	91.1 ^a^ ± 13.5
Crude protein ^2,3^ (g/head/day)	823 ^c^ ± 57.7	1177 ^b^ ± 123	1283 ^a^ ± 190
Crude fat (g/head/day)	248 ^c^ ± 17.6	364 ^a^ ± 37.9	316 ^b^ ± 46.6
Crude fiber (g/head/day)	260 ^c^ ± 18.1	384 ^a^ ± 40.2	339 ^b^ ± 50.1

^a,b,c^ Means in a row with different superscripts differ significantly (*p* < 0.05). ^1^ CON: Control diet; AL: ad libitum feeding without L-arginine; AL+Arg: ad libitum feeding supplemented with 1% L-arginine. ^2^ Crude protein (CP) was calculated as N × 6.25. As L-arginine has a high nitrogen content, CP is slightly overestimated in the AL+Arg group, which should be considered when interpreting nutrient intake data. ^3^ According to the recommendations for lactating sows with a suckling period of 25 days made by the German Society for Nutrition Physiology [39], the required amino acid supply is expressed as precaecally digestible crude protein. Since the present study reports CP without digestibility correction, a direct quantitative comparison with the German Society for Nutrition Physiology reference values is not possible and will be addressed in the Discussion.

**Table 6:** Body weight, backfat thickness, and backfat thickness loss in sows.

Parameter		CON ^1^ (*n* = 24)	AL ^1^ (*n* = 26)	AL+Arg ^1^ (*n* = 21)
Body weight (kg)	At entry to farrowing compartment	281 ^Aa^ ± 23.0	292 ^Aa^ ± 23.9	279 ^Aa^ ± 22.4
	At weaning	230 ^Bb^ ± 21.3	254 ^Ba^ ± 25.2	247 ^Ba^ ± 21.2
Backfat thickness (mm)	At entry to farrowing compartment	19.9 ^Aa^ ± 2.72	20.2 ^Aa^ ± 2.75	20.5 ^Aa^ ± 5.36
	At weaning	15.2 ^Bb^ ± 1.93	17.3 ^Ba^ ± 2.92	17.9 ^Ba^ ± 4.56
Backfat thickness loss	Absolute (mm)	4.71 ^a^ ± 2.16	2.96 ^b^ ± 1.91	2.62 ^b^ ± 1.12
	Relative (%) ^2^	23.1 ^a^ ± 9.06	14.6 ^b^ ± 9.30	12.6 ^b^ ± 3.75
Calculated milk production (kg)	During lactation	260 ^a^ ± 43.0	287 ^a^ ± 57.8	288 ^a^ ± 73.5

^a,b^ Values within a row with different superscripts differ significantly at *p* < 0.05. ^A,B^ Values within a column with different superscripts differ significantly at *p* < 0.05. ^1^ CON: Control diet; AL: ad libitum feeding without L-arginine; AL+Arg: ad libitum feeding supplemented with 1% L-arginine. ^2^ Before start of lactation.

**Table 7:** Reproduction performance parameters of sows.

Item	CON ^1^	AL ^1^	AL+Arg ^1^
Reproductive performance of the initial litter ^2^			
Total born piglets	13.7 ^a^ ± 3.09	14.5 ^a^ ± 3.52	14.1 ^a^ ± 3.12
Live born piglets	13.3 ^a^ ± 2.94	13.8 ^a^ ± 3.40	13.7 ^a^ ± 3.48
Stillborn piglets	0.42 ^a^ ± 0.93	0.69 ^a^ ± 1.09	0.38 ^a^ ± 0.81
Reproduction performance in follow-up litter ^3^			
Total born piglets	15.7 ^a^ ± 2.82	15.9 ^a^ ± 3.77	13.5 ^a^ ± 4.10
Stillborn piglets	0.50 ^a^ ± 0.91	0.68 ^a^ ± 1.36	1.12 ^a^ ± 2.55

^a^ Values within a row with similar superscripts do not differ significantly at *p* < 0.05. ^1^ CON: Control diet; AL: ad libitum feeding without L-arginine; AL+Arg: ad libitum feeding supplemented with 1% L-arginine. ^2^ CON (*n* = 24); AL (*n* = 26); AL+Arg (*n* = 21). ^3^ CON (*n* = 22); AL (*n* = 22); AL+Arg (*n* = 17).

**Table 8:** Suckling period, body weight of piglets 24 h postpartum and at weaning, and litter growth depending on the feeding regime.

Item	CON ^1^	AL ^1^	AL+Arg ^1^
Body weight of piglets 24 h postpartum (kg)	1.58 ^a^ ± 0.39 (*n* = 280)	1.49 ^b^ ± 0.31 (*n* = 309)	1.48 ^b^ ± 0.35 (*n* = 229)
Suckling period (day)	27.0 ^a^ ± 2.93	27.3 ^a^ ± 1.50	27.1 ^a^ ± 1.61
Number of piglets per sow after litter standardization (*n*)	11.7 ^a^ ± 1.55	11.9 ^a^ ± 1.80	10.9 ^a^ ± 2.00
Number of weaned piglets per sow (*n*)	11.5 ^a^ ± 1.62	11.5 ^a^ ± 1.94	10.9 ^a^ ± 1.81
Pre-weaning mortality (%)	2.50 ^a^	7.40 ^a^	3.90 ^a^
Body weight of piglets at weaning (kg)	7.41 ^c^ ± 1.54 (*n* = 275)	8.03 ^b^ ± 1.77 (*n* = 287)	8.32 ^a^ ± 1.67 (*n* = 229)
Litter growth (kg)	66.9 ^a^ ± 10.7	74.3 ^a^ ± 14.8	73.8 ^a^ ± 18.1
Amount of prestarter used per litter (kg)	4.00 ^a^ ± 0.693	4.62 ^a^ ± 1.61	4.11 ^a^ ± 0.981

^a,b,c^ Values within a row with different superscripts differ significantly at *p* < 0.05. ^1^ CON: Control diet; AL: ad libitum feeding without L-arginine; AL+Arg: ad libitum feeding supplemented with 1% L-arginine.

**Table 9:** Daily weight gain (kg) of suckling pigs depending on birth weight class.

Birth Weight Class	CON ^1^	AL ^1^	AL+Arg ^1^
≤1.00	0.18 ^b^ ± 0.03	0.18 ^b^ ± 0.05	0.23 ^a^ ± 0.04
>1.00–1.25	0.21 ^b^ ± 0.04	0.21 ^b^ ± 0.04	0.25 ^a^ ± 0.05
>1.25–1.50	0.22 ^b^ ± 0.04	0.24 ^a^ ± 0.05	0.25 ^a^ ± 0.05
>1.50–1.75	0.22 ^b^ ± 0.05	0.26 ^a^ ± 0.05	0.26 ^a^ ± 0.05
>1.75–2.00	0.25 ^b^ ± 0.04	0.27 ^a^ ± 0.05	0.27 ^a^ ± 0.05
>2.00	0.23 ^b^ ± 0.04	0.30 ^a^ ± 0.05	0.30 ^a^ ± 0.04

^a,b^ Values within a row with different superscripts differ significantly at *p* < 0.05. ^1^ CON: Control diet; AL: ad libitum feeding without L-arginine; AL+Arg: ad libitum feeding supplemented with 1% L-arginine.

**Table 10:** Lactation feed intake from day 3 postpartum until weaning (kg in dry matter/day) of sows by backfat thickness category at entry to the farrowing compartment.

Backfat Category	AL ^1^	AL+Arg ^1^
<19 mm	6.50 ^a^ ± 0.44	6.20 ^a^ ± 0.79
19–23 mm	6.40 ^a^ ± 0.62	6.50 ^a^ ± 0.78
>23 mm	5.30 ^b^ ± 0.55	5.10 ^b^ ± 0.81

^a,b^ Values within a column with different superscripts differ significantly at *p* < 0.05. ^1^ AL: ad libitum feeding without L-arginine; AL+Arg: ad libitum feeding supplemented with 1% L-arginine.

## Data Availability

The data presented in this study are available in this manuscript.

## Supplementary Materials

The following supporting information can be downloaded at: https://www.mdpi.com/article/10.3390/ani16172687/s1, Table S1: Composition of the vitamin–mineral premix (per kg premix, as-fed basis) included in the supplement used in pregnant and lactating sow diets; the supplement was incorporated at 10% as-fed basis.
