# Supplementary material for: Effects of Ad Libitum Feeding and Arginine Supplementation on Growth of Piglets and Reproductive Performance Parameters of Sows
Source: Animals (Basel). 2026 Aug 28;16(17):2687. doi: 10.3390/ani16172687 (PMC13564311; doi:10.3390/ani16172687)
Supplement: Supplementary file 1 [file animals-16-02687-s001.zip › animals-4487797-supplementary.pdf]

Supplementary Table S1. Composition of the vitamin–mineral premix (per kg premix, as-fed basis) included in the supplement used in pregnant and lactating sow diets; the supplement was incorporated at 10% as-fed basis.

**Ingredient**	**Pregnant diet**	**Lactating diet**	**Unit**
Vitamin A	450,000	325,000	IU
Vitamin D3	75,000	56,000	IU
Vitamin E	4,000	4,000	mg
Vitamin C	2,500	4,000	mg
Vitamin K3	200	200	mg
Vitamin B1	140	120	mg
Vitamin B2	350	300	mg
Vitamin B6	240	200	mg
Vitamin B12	2,400	2,000	μg
Niacin	1,400	1,200	mg
Calcium-D-pantothenate	800	700	mg
Folic acid	100	100	mg
Biotin	30,000	20,000	μg
Choline chloride	40,000	28,500	mg
Iron	3,000	3,000	mg
Zinc	3,500	3,000	mg
Manganese	3,000	1,500	mg
Copper	600	425	mg
Iodine	100	50	mg
Selenium	16	10	mg
L-Carnitine	4,000	1,650	mg

Premix supplied by Invaso GmbH, Ergoldsbach, Germany.
